# Tetanus following replantation of an amputated finger: a case report

**DOI:** 10.1186/1752-1947-6-343

**Published:** 2012-10-08

**Authors:** Kenji Hayashida, Chikako Murakami, Masaki Fujioka

**Affiliations:** 1 Division of Plastic and Reconstructive Surgery, National Nagasaki Medical Center, 1001-1 Kubara 2, Ohmura City, 856-8562, Japan

**Keywords:** Exposure of neurovascular bundle, Replantation of an amputated finger, Tetanus

## Abstract

**Introduction:**

Tetanus is an infectious disease caused by tetanus toxin produced by *Clostridium tetani* and induces severe neurological manifestations. We treated a patient who developed tetanus during hospitalization for replantation of an amputated finger. To the best of our knowledge, this is the first published case report of such an entity.

**Case presentation:**

A 49-year-old Japanese man had an amputation of his right middle finger at the distal interphalangeal joint region in an accident at work. His middle finger was successfully replanted, but his fingertip was partially necrotized because of crushing and so additional reconstruction with a reverse digital arterial flap was performed 15 days after the injury. Tetanus developed 21 days after replantation of the middle finger, but symptoms remitted via rapid diagnosis and treatment.

**Conclusions:**

In replantation after finger trauma with exposure of nerve and blood vessel bundles, concern over injuring nerves and blood vessels may prevent irrigation and debridement from being performed sufficiently; these treatments may have been insufficiently performed in this patient. It is likely that the replanted middle finger partially adhered, and *Clostridium tetani* colonized the partially necrotized region. Even when there is only limited soil contamination, administration of tetanus toxoid and anti-tetanus immunoglobulin is necessary when the fingers are injured outdoors and the finger nerves and blood vessels are exposed. The drugs should be administered just after replantation if the finger has been amputated. However, if clinicians pay attention to the possibility of tetanus development, treatment can be rapidly initiated.

## Introduction

Tetanus is an infectious disease caused by tetanus toxin produced by *Clostridium tetani* and induces severe neurological manifestations, such as tonic convulsions. The incidence and mortality rate have recently decreased because of the dissemination of tetanus toxoid vaccination and improvements in intensive care. Here, we report what to the best of our knowledge is the first case of tetanus to develop after replantation of an amputated finger and discuss the prevention of tetanus after trauma of the fingers.

## Case presentation

**Figure 1:**
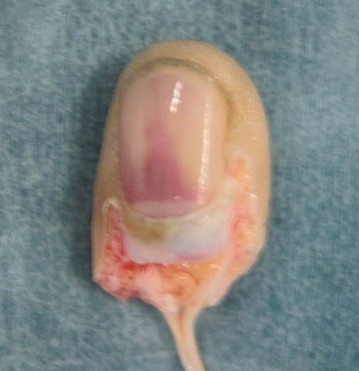
Amputated middle finger shortly after the time of injury.

**Figure 2:**
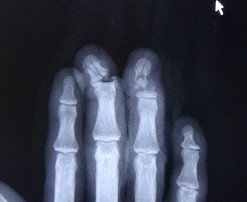
X-ray of right hand shortly after the time of injury.

A 49-year-old Japanese man whose gloved hand was caught between a gas cylinder and a concrete floor was taken to our hospital by ambulance. His right middle finger had been amputated in the distal interphalangeal (DIP) joint region, and his right ring finger was connected only by nerve and blood vessel bundles, showing a compound fracture of the distal phalanx (Figures 1 and 2). Our institute’s approach in cases of trauma is to inject tetanus toxoid and human anti-tetanus immunoglobulin (TIG) if the wound is dirty. In this case, no tetanus toxoid was injected since there was no soil contamination. The bone was comminuted, and the crushing was found to be severe, but replantation was considered appropriate, and replantation of the middle finger and osteosynthesis of the distal phalanx of the ring finger were performed on the day that our patient was injured.

Surgery was performed under axillary block anesthesia. After irrigation with 500mL of warm saline, the DIP joint of the middle finger was fixed, and the ulnar-side finger artery and dorsal cutaneous vein were anastomosed. In the ring finger, the distal phalanx was reduced and fixed. Minimal debridement was applied to only a part of the skin.

**Figure 3:**
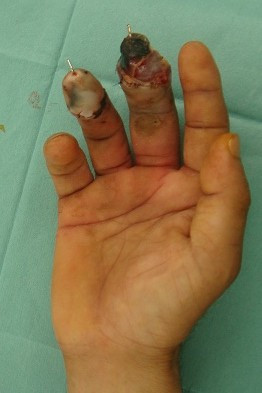
The phalangeal region of the middle finger was partially necrotized.

**Figure 4:**
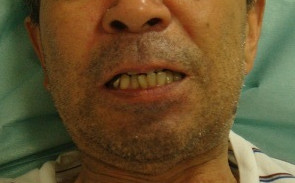
Maximum mouth opening when trismus developed.

Our patient was admitted for observation. There were no infectious signs in his hand. His middle finger was successfully replanted, but his fingertip was partially necrotized because of crushing (Figure 3), and so additional reconstruction with a reverse digital arterial flap was performed 15 days after injury. The wound of his ring finger healed completely 18 days after the injury. No particular symptoms developed and there was no problem with the condition of the flap, but aggravation of lower back pain, trismus, and convulsion suddenly occurred 21 days after the injury (Figure 4). The wound of his middle finger was immediately inspected, and discharge of a whitish turbid exudate from the region around the flap was noted. The wound was opened and irrigated with a large volume of saline. No bacterium was detected on exudate culture, and isolation or identification was not possible. On the basis of the clinical symptoms, tetanus was diagnosed and treatment was initiated. TIG (6000 units) was intravenously administered on the day of onset, and 6000 units of TIG and 0.5mL of intramuscular tetanus toxoid were administered the following day. We administered treatment with penicillin antibiotics. However, the convulsions did not remit and, in fact, slowly became aggravated. Thus, tracheal intubation was performed, and our patient underwent artificial respiratory management. Anticonvulsant and sedative were concomitantly administered, but convulsion was readily induced by light stimulation, such as irrigation of the wound. The frequency and intensity of convulsive seizures started to decrease slightly at about 10 days after onset, and a tapering of the intravenous anticonvulsant injection was initiated. Our patient was weaned from the ventilator 12 days after onset. The distal phalanx fracture of his ring finger healed 6 weeks after the injury. Conservative treatment of the open wound of his middle finger was continued, and the wound healed 8 weeks after the injury. The fingertip morphology of his middle finger was relatively favorable, but owing to rest for tetanus treatment, rehabilitation could not be performed, and joint contracture remained in his right middle and ring fingers. No systemic problem occurred afterward, and our patient was discharged 12 weeks after the injury.

## Discussion

*C. tetani* is widely present in soil, and its infection occurs through open wounds. The wound of this patient was not contaminated with soil given that the work was not muddy and he wore gloves, but toxoid and TIG should have been actively administered on the assumption that basic immunity was lacking. Because of an antibody titer retention of only about 10 years, the positivity rate of the anti-tetanus antibody titer markedly decreases after 40 years of age in Japan [1]. Many cases of tetanus caused by minor traumas and gardening have been reported, and tetanus development is impossible to predict from the severity of the trauma [2-6]. However, it is possible to reduce the incidence by irrigation and debridement of the injured regions [6,7] because *C. tetani* adheres mainly to necrotizing tissue. In this patient, it is likely that the replanted middle finger only partially engrafted and that *C. tetani* colonized the partially necrotized region. Because the amputated finger was separated from our patient, the prophylaxis of toxoid and TIG would not have been appropriate just after the injury. If we had administered toxoid and TIG just after replantation, we could have prevented tetanus in this patient.

*C. tetani* is difficult to isolate or identify [8,9], but diagnosis by bacterial culture or serological testing is not necessary. Even if the bacterium can be isolated, several days are required to obtain the results. Thus, when symptoms such as trismus and systemic convulsions develop after trauma, tetanus should be actively suspected and rapid diagnosis and treatment should be initiated. Because our patient was admitted, tetanus could be diagnosed and treated relatively early, and he could resume his life with no serious complications or sequelae.

In replantation after finger traumas or the treatment of a compound fracture with exposure of nerve and blood vessel bundles, concern over injuring nerves and blood vessels may prevent irrigation and debridement from being performed sufficiently, and these treatments may have been insufficiently performed in this patient. Even when only limited soil contamination has occurred, administration of tetanus toxoid and TIG is necessary just after replantation of the fingers when the fingers are injured outdoors and the finger nerves and blood vessels are exposed. However, if clinicians pay attention to the possibility of tetanus development, treatment can be rapidly initiated.

## Conclusions

We encountered a patient in whom tetanus developed after replantation of an amputated finger. It is necessary to pay attention to the possibility of tetanus development after trauma to the fingers. Although prevention is important for tetanus, the outcomes can be improved by early diagnosis and treatment.

## Consent

Written informed consent was obtained from the patient for publication of this case report and any accompanying images. A copy of the written consent is available for review by the Editor-in-Chief of this journal.

## Abbreviations

DIP: distal interphalangeal; TIG: tetanus immunoglobulin.

## Competing interests

The authors declare that they have no competing interests.

## Authors’ contributions

CM and MF reviewed the paper and suggested changes. All authors read and approved the final manuscript.
